# Assessing land use and land cover changes and agricultural farmland expansions in Gambella Region, Ethiopia, using Landsat 5 and Sentinel 2a multispectral data

**DOI:** 10.1016/j.heliyon.2018.e00919

**Published:** 2018-11-08

**Authors:** Azeb W. Degife, Florian Zabel, Wolfram Mauser

**Affiliations:** Department of Geography, Ludwig-Maximilians-University, Luisenstr. 37, 80333 Munich, Germany

**Keywords:** Environmental science, Earth sciences, Geography

## Abstract

The pace of change in land use and cover in Ethiopia depends on three main factors that cause pressure on agriculture land: resettlement programmes, population growth and increasing agricultural investments. Gambella is one of the regions of Ethiopia that attracts large-scale agricultural investments that extensively drive land use and cover changes in the region. The aim of this study is to examine the rate, extent and distribution of various land use and cover changes in Gambella Regional State, Ethiopia, from 1987 to 2017. The analysis is mainly based on Landsat 5 and Sentinel 2A satellite images and fieldwork. Two Landsat Thematic Mapper and a Sentinel 2A image were used for determining the maximum likelihood of land use/cover classification. The results show that farmland decreased by 26 km^2^ from 1987 to 2000; however, during the last two decades, agricultural land area increased by 599 km^2^, mainly at the cost of tropical grasslands and forests. We found that areas cultivated by smallholder farmers increased by 9.17% from 1987 to 2000. However, small-scale farm activities decreased by 7% from 2000 to 2017. Areas cultivated by large-scale state farms totalled 202 km^2^ in 1987; but by 2000, this large-scale state farming had been completely abandoned by the state, and as a result, its land use has decreased to zero. Despite this, in 2017 large-scale farming increased to 746 km^2^. In addition, Gambella National Park, which is the nation's largest national park and ecosystem, was also largely affected by Land Use and Land Cover changes. The conversion of savannah/tropical grasslands to agricultural farmland has caused varied and extensive environmental degradation to the park. The Land Use and Land Cover changes in the Gambella region are discussed on the basis of underlying socioeconomic factors.

## 1 Introduction

Land Use and Land Cover changes (LULCC) involves either a shifting to a different land use or an intensification of existing land [1]. Currently, across the world, an increasing demand for space for settlement, agricultural investment and industrial activities is being observed [2, 3]. This leads to unprecedented LULCC, and these have caused both socioeconomic and environmental problems [4]. Human use of land has had a profound effect upon the natural environment resulting in an observable pattern in Land Use and Land Cover (LU/LC) over time [5, 6]. The aim of this paper is to explore the dynamics and mechanisms of LULCC during the last 30 years in the Gambella region in the southwest of Ethiopia, where the LU/LC dynamics are the largest in the country [7, 8].

In Ethiopia and especially in the Gambella region, the pace of change in LU/LC depends on three main factors: resettlement, population growth and increasing agricultural land pressure [8, 9, 10]. All three factors have own contribution for the LULCC. Between 1983 and 1985, the worst famine in the country's history led to the deaths of more than 400,000 people. After this drought, the Ethiopian government, Derg (the Coordinating Committee of the Armed Forces, Police and Territorial Army that ruled Ethiopia from 1974 to 1991), made and carried out resettlement plans to relocate the rural population with the principal strategy of ensuring food security [11]. In the mid-1980s, nearly 800,000 people were relocated, mostly from the northern highlands to distant areas in the southwest, including the Gambella region [12]. As a result, LULCC have heavily modified the natural landscape of the Gambella region, and large areas across the region have been deforested and/or drained [13]. Similarly, in the 2000s the Federal Democratic Republic of Ethiopia (FDRE) took the voluntary villagization scheme (VVS) as a strategy to transform the livelihood of settlers and ensure food security by providing socioeconomic and infrastructural delivery, and solving the problems created by the Derg on a voluntarily basis through an intra-regional approach. This resettlement programme, that has been ongoing since the 1980s, has caused a large shift in population into the Gambella region. According to the Ethiopian Central Statistical Agency (2015), the total population of the Gambella region rose from 182,000 in 1994, to 307,000 in 2007, and to 409,000 in 2015. This rising population over the years has created considerable pressure on the land and its natural resources, including its forests and water [14].

The Gambella region has both a unique ecology and an extraordinarily rich biodiversity. At the same time, the level of resource use in the Gambella region is still comparatively low. This explains why the main mechanism of LULCC in the Gambella region is agricultural land expansion [8]. Besides the spread of small-scale farming as a result of the population increase, one of the main agricultural development policies is large-scale farming investment. It is promoted by the Ethiopian government as part of an infrastructure expansion and economic stabilisation programme. Since 2005, the country, as well as the Gambella region, saw a surge of domestic and foreign investment into commercial farming. From the late 1990s to the end of 2008, almost 3.5 million hectares of land were transferred to both domestic and foreign investors, mainly in the southwestern part of Ethiopia [8]. Because of its favourable environmental conditions, the Gambella region has attracted large-scale agricultural investment. During the same period, both the federal and regional government have awarded 1.1 million hectares of suitable farmland in the Gambella region to foreign and local companies/investors. Since 2008, Gambella has become the major target point for foreign investors.

These developments, which began in the 1980s and are still ongoing, have a major impact on the magnitude and nature of LULCC in the Gambella region and throughout the whole of Ethiopia. It is therefore highly relevant to assess and understand LULCC in this region. Existing studies so far have mainly focused on either deforestation or LULCC in specific districts of the Gambella region [13].

A complete picture of LULCC in the entire Gambella region, from the mid-1980s to the present day, does not exist to the best of the authors' knowledge. Therefore, the overall objective of this paper is to examine the rate, extent and distribution of various LULCC coverages in Gambella Regional State. This also includes a contribution, as a first step, to documenting LU intensity changes in the region by identifying and analysing the rate of small-scale and commercial agricultural land expansions and their overall extent.

## 2 Materials and methods

### 2.1 Study area

**Fig. 1:**
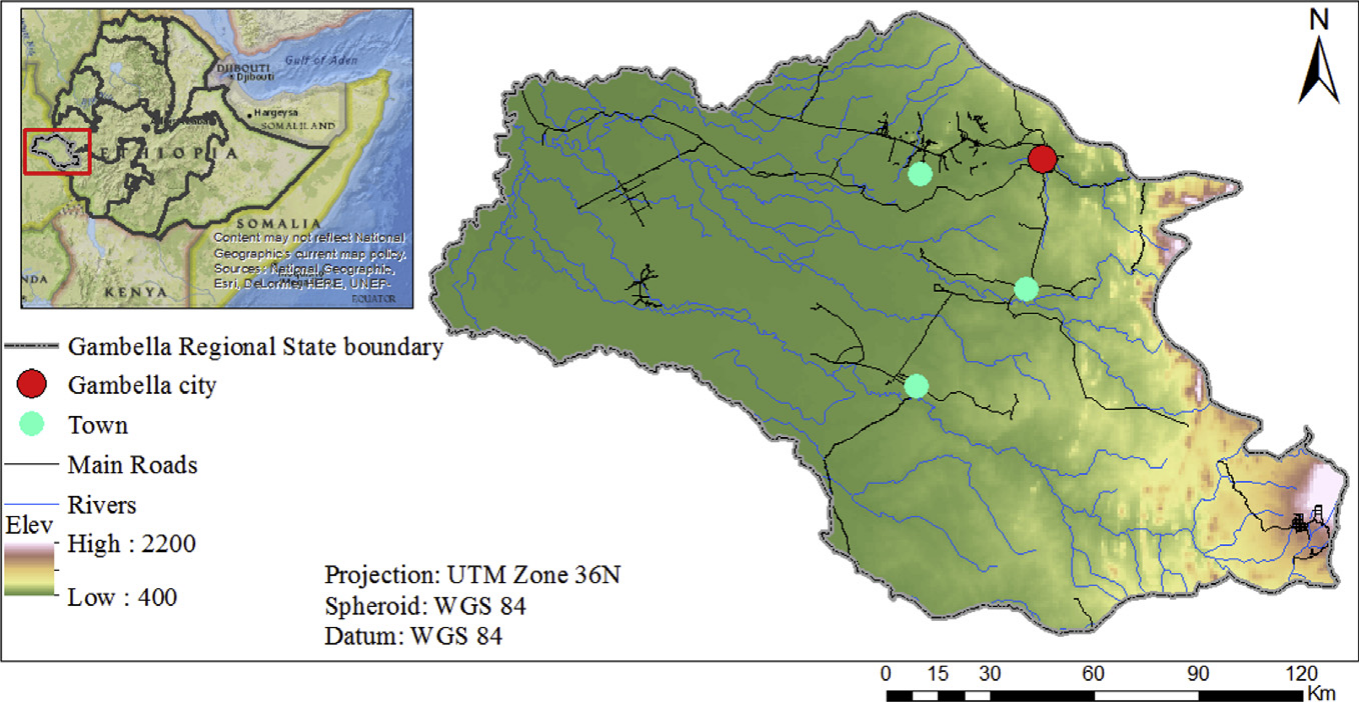
Locational map: Federal Democratic Republic of Ethiopia and Regional States boundary (left) and Gambella Regional State (right).

Gambella region is one of nine administrative regions in the western part of Ethiopia (see Fig. 1). The region covers a total area of 25,521 km^2^ [15]. It is located between 6°28′38″ to 8°34′ North Latitude and 33° to 35°11′11″ East Longitude. As shown in Fig. 1, it borders two other Ethiopian regions – Oromia to the north and east and the Southern Nations, Nationalities and Peoples' Regional State (SNNPRS) to the south. To the west it shares a border with South Sudan. The region is comprised of three administrative zones (Anyuak, Nuer and Majang) and 13 districts (woredas), one special district and one city administration [16]. Gambella is one of the emerging regions in Ethiopia. Its economy is predominantly based on agriculture with mixed farming among the Anyuak and Majang people and agro-pastoral among the Nuer people [17]. The region lacks infrastructure with a poor transportation network among the districts (woredas).

The Gambella region lies within the hot to warm humid lowland agro-ecological zone. The topography divides the Gambella region into two broad sub-regions, which are between 900 to 2200 masl (metres above sea level) and the flood plains below 500 masl. Its climate is classified as tropical savannah (Aw) by Köppen and Geiger [18] with an average temperature of 27.6 °C and 1,197 mm of average annual precipitation [19]. The rainy season starts at the end of April and lasts until October with the maximum rainfall in July [20]. In addition, there are two main harvesting times known as Meher and Belg. In the Meher, crops are harvested from September to February and in the Belg, crops are harvested from March to August. Baro, Gilo, Akobo, and Alwero are the main rivers crossing the region [21]. The Gambella region, due to favourable soil, topography and climate conditions, is known to be one of the most fertile regions in Ethiopia and is suitable for growing various types of crops. The region has very high crop suitability compared to other parts of Ethiopia [22]. Crop suitability, abundant water resources, unused/underutilised land and Ethiopian government policy (Agriculture Development Led Industrialisation) are the main facilitating factors for the expansion of large-scale commercial farming in the region.

### 2.2 Remote sensing data processing and analysis

In order to analyse LULCC in the Gambella region, medium resolution satellite remote sensing data from the Landsat and SENTINEL-2 sensors were selected, covering the period from the mid-1980s to the present. Multi-temporal satellite imagery plays a vital role in quantifying spatial and temporal LULCC in the area [23]. LULCC maps were determined from the selected images through a number of consecutive digital processing steps ranging from image selection and download, pre-processing, classification using known training sites, validation using a separate set of known validation sites, classification post-processing and map as well as change map production. The spatial map resolution is 30 m in the case of Landsat TM and 10 m in the case of Sentinel-2 images [24] (https://earthexplorer.usgs.gov/). This medium resolution satellite imagery offers the potential for an accurate land cover classification and pattern analyses [25] for the detection and quantification of historical LULCC, especially in remote regions [26] such as the Gambella region where not many data on present LULCC are currently available. Multispectral satellite images of the region were selected for the three years of 1987, 2000 and 2017. Care was taken to select the images to match their phenological periods as closely as possible in order to note the changes in vegetation development. At the same time the images acquired were from the period immediately after the dry season in order to be able to observe the whole range of active vegetation and to avoid investigating dried up vegetation, which cannot be separated into LULCC categories. This being a vital prerequisite for detecting the full range of categories and later comparison of the spatial extent of the classified LULCC categories, it nevertheless limits the number of available images. Based on these considerations Landsat and Sentinel 2a images from January and February 1987, 2000 and 2017 respectively were selected and downloaded (as illustrated in Table 1, all the listed images from each year were mosaicked and then clipped based on the Gambella feature, before being processed and classified).

**Table 1:** Dates and scene ID number of Landsat 5 and Sentinel 2a images used.

Year	Day and month	Scene/tile	Entity ID
1987	13/Jan	171/054	LT05_L1TP_171054_19870113_20170215
	13/Jan	171/055	LT05_L1TP_171055_19870113_20170215
	20/Jan	172/054	LT05_L1TP_172054_19870120_20170215
	20/Jan	172/055	LT05_L1TP_172055_19870120_20170215
	22/Jan	170/055	LT05_L1TP_170055_19870122_20170215
2000	01/Jan	171/054	LT05_L1TP_171054_20000101_20170908
	01/Jan	171/055	LT05_L1TP_171055_20000101_20170908
	24/Jan	172/054	LT05_L1TP_172054_20000124_20171214
	24/Jan	172/055	LT05_L1TP_172055_20000124_20171214
	27/Feb	170/055	LT05_L1GS_170055_20000227_20171214
2017	05/Jan	T36NWP	S2A_MSIL1C_20170105T080312_N0204_R035_T36NWP_20170105T081256.SAFE
	05/Jan	T36NYP	S2A_MSIL1C_20170105T080312_N0204_R035_T36NYP_20170105T081256.SAFE
	05/Jan	T36PWQ	S2A_MSIL1C_20170105T080312_N0204_R035_T36PWQ_20170105T081256.SAFE
	15/Jan	T36NXN	S2A_MSIL1C_20170115T080251_N0204_R035_T36NXN_20170115T081421.SAFE
	15/Jan	T36NXP	S2A_MSIL1C_20170115T080251_N0204_R035_T36NXP_20170115T081421.SAFE
	15/Jan	T36NYN	S2A_MSIL1C_20170115T080251_N0204_R035_T36NYN_20170115T081421.SAFE
	15/Jan	T36PXQ	S2A_MSIL1C_20170115T080251_N0204_R035_T36PXQ_20170115T081421.SAFE
	15/Jan	T36PYQ	S2A_MSIL1C_20170115T080251_N0204_R035_T36PYQ_20170115T081421.SAFE

The second step of digital image processing consisted, after radiometric image enhancement techniques of the Landsat TM data [27], of a two-stage image classification procedure that included unsupervised Iterative Self Organising Data Analysis (ISODATA) and supervised (Maximum Likelihood) classification [28]. Unsupervised (ISODATA) clustering algorithms determined the characteristics of the natural groupings of cells in multidimensional attribute space [29]. The unsupervised ISODATA algorithm created 25 clusters of pixels using ten different iterations and a convergence threshold of 0.99. From the 25 clusters, seven separate LU/LC classes were merged for the years 1987, 2000 and 2017 based on their extracted spectral signatures. The delineated LU/LC classes were: water bodies, man-made structures, farmland, forest land, tropical grasslands, wetland vegetated area and barren or sparsely vegetated land. These classes are described in Table 2 based on Corine LU/LC classes. This unsupervised classification of LU/LC classes was then used as a basis for the following supervised classification.

**Table 2:** Description of the LU/LC classes.

	Land cover	Description
1	Water bodies	This class of land cover describes areas covered with water including rivers and lakes
2	Artificial areas	This class describes the land covered with buildings in the study area. It includes commercial, residential, industrial and transportation infrastructure.
3	Farmland	This class describes land which is mainly used for growing crops. Crops in this land are either grown by irrigation (commercial farmland) or are rain-fed (small-scale farmland).
4	Forest land	This class describes the areas with evergreen trees, mainly growing naturally.
5	Tropical grasslands	This class of land cover defines grassland or tropical savannah as the main vegetation cover.
6	Wetlands	This class describes areas with swampy or marshland vegetation.
7	Barren or sparsely vegetated land	This describes the land left without vegetation cover. This can result from abandoned cropland and eroded land due to degradation, exposed soil, sand or rocks,

Supervised classification is based on training sites where land use is known and where signatures can be used to train the algorithm. For the years 1987 and 2000, no supervised classification training sites with known LU/LC were available. The training sites were therefore selected based on the spectral signature of the classes and, in the case of known stable land uses, through comparison with the known land uses in 2017. For each of the predetermined LU/LC types from the unsupervised approach, training sites were selected and spectral signatures derived. In general, a satisfactory spectral signature is one ensuring that there is ‘minimal confusion’ among the LU/LC classes to be mapped [30] and at the same time covering the spectral variability of the class.

In 2017, through simple random sampling the ground truth data was collected for the supervised classification. This randomness of ground truth data ensures that all parts of the study area have an equal chance of being sampled. Therefore, training sites were identified based on field-collected data using Global Positioning System (GPS), on-field checks of the high-resolution January 2017 Sentinel 2a images, and familiarity with the area. The spectral signatures of the selected LU/LC classes represented by their training areas were then used to produce a Maximum Likelihood Classification of the satellite images. The number of training sites varied from one LU/LC class to another, depending on ease of identification and the level of variability of pixel values within the site. For instance, due to spectrally similarities in water and forest areas, the training sites were set to 60 per class. Whereas, both in farmlands and tropical grasslands, the spectral variability were higher, as result the training site per class were set to 120 and 100 respectively. For more accurate results several training sites for each class located in different parts of the image. The Maximum Likelihood Classification is the most widely used per-pixel method. In addition to the reflectance values, it also takes into account the covariance of the information contained in the sensors' spectral bands of LU/LC classes [31].

### 2.3 Post classification and accuracy assessment

Post-classification refinement was conducted in order to improve classification accuracy and to reduce the number of erratic misclassifications. After classification, ground verification was done in order to check the accuracy of the classified LU/LC map [32]. Based on the ground verification, necessary corrections and adjustments were made. To determine the accuracy of the 1987 and 2000 classifications, a total of 760 stratified randomly generated points (locations) were selected in the classified image of the study area (as shown in Tables 3 and 4). Google Earth Time Lapse was used as the main reference source to identify these selected points. Accuracy assessment of the 2017 classification of the Sentinel 2a satellite images was based on ground truth data collected during the 2017 fieldtrip to the Gambella region. In addition, a total of 560 points (locations) were identified in the classified images of the study area (Table 5). Google Earth was used as a reference source to assess these selected points. The overall accuracy was calculated by dividing the sum of the correctly classified sample points by the total number of sample points.

**Table 3:** Error matrix accuracy totals for the classified image (1987).

Class types determined from reference source										
Year: 1987		Water bodies	Artificial areas	Farmland	Forest land	Tropical grasslands	Wetland vegetated area	Barren or sparsely vegetated area	Classification overall	Producer accuracy % (Precision)
Class types determined from classified map	Water bodies	73	0	0	0	23	0	0	96	73
	Artificial areas	0	27	26	0	2	0	0	55	50
	Farmland	0	2	88	0	19	1	0	110	80
	Forest land	0	0	0	74	0	0	0	74	100
	Tropical grasslands	0	2	37	0	264	0	1	304	87
	Wetland vegetated area	0	0	0	0	1	53	0	54	98
	Barren or sparsely vegetated area	0	0	9	0	1	5	52	67	78
	Truth overall	73	31	160	74	310	59	53	760	
	User accuracy % (Recall)	100	87	55	100	85	85	98		
Overall accuracy = 82.79%										
Kappa = 0.776

**Table 4:** Error matrix accuracy totals for the classified image (2000).

Class types determined from reference source										
Year: 2000		Water bodies	Artificial areas	Farmland	Forest land	Tropical grasslands	Wetland vegetated area	Barren or sparsely vegetated area	Classification overall	Producer accuracy % (Precision)
Class types determined from classified map	Water bodies	108	0	0	0	0	0	0	108	100
	Artificial areas	0	104	0	0	7	0	4	115	90
	Farmland	0	0	108	0	4	0	0	112	96
	Forest land	0	0	0	108	4	11	0	123	88
	Tropical grasslands	0	0	4	0	93	25	25	147	63
	Wetland vegetated area	0	0	0	0	0	72	0	72	100
	Barren or sparsely vegetated area	0	4	9	0	0	0	79	83	95
	Truth overall	108	108	112	108	108	108	108	760	
	User accuracy % (Recall)	100	87	55	100	85	85	98		
Overall accuracy = 88.4%										
Kappa = 0.865

**Table 5:** Error matrix accuracy totals for the classified image (2017).

Class types determined from reference source										
Year: 2017		Water bodies	Artificial areas	Farmland	Forest land	Tropical grasslands	Wetland vegetated area	Barren or sparsely vegetated area	Classification overall	Producer accuracy % (Precision)
Class types determined from classified map	Water bodies	76	2	1	1	0	0	0	80	95
	Artificial areas	0	78	1	0	0	0	1	80	97
	Farmland	0	0	62	1	0	2	15	80	78
	Forest land	0	0	0	80	0	0	0	80	100
	Tropical grasslands	0	0	1	0	78	0	1	80	98
	Wetland vegetated area	0	0	0	0	0	80	0	80	100
	Barren or sparsely vegetated area	0	4	2	0	4	1	73	80	91
	Truth overall	76	80	67	82	82	83	90	560	
	User accuracy % (Recall)	100	97	92	98	95	96	81		
Overall accuracy = 94.4%										
Kappa = 0.931										

### 2.4 LULCC detection

LULCC in the region was detected and assessed, and the size and distribution of the altered areas were quantified. The map from the validated classification in 1987 and 2000 using Landsat TM data was compared with the map produced for 2017 using Sentinel 2a, and then a complete matrix of categorical change was obtained. The Sentinel 2 classification result was re-projected and resampled from 10 m to the same 30 m resolution than the classification obtained by Landsat using a majority filter. This allows for comparison of Sentinel and Landsat images at the costs of spatial information of the Sentinel image. Finally, the 1987, 2000 and 2017 supervised classification results were compared using cross-tabulation to quantitatively determine the LU/LC dynamics. A pixel-based comparison was also used to produce quantitative change information.

### 2.5 Methods to classify large-scale commercial and small-scale farmland

The classification results say nothing about farming intensity. Therefore we chose a different approach for the distinction of farming intensity. It is based on the assumption that both the crop type selection and the level of farming intensity in terms of seed selection, use of machinery and fertiliser, as well as pesticide application, is related to field size. Mechanised agriculture allows and demands large field sizes to produce commodities at competitive prices. On the other hand, smallholders without market access and mechanisation cannot manage to work large fields. We masked farmland class from selected satellite imagery from each year and then separated large-scale farmland from small-scale farmland based on land holding size. In addition, high-resolution satellite images (Sentinel 2a) and Google Earth were used as a reference to distinguish small-scale and large-scale commercial farmlands. Finally, depending on a manual digitisation approach on the selected satellite imagery, we extracted large- and small-scale agricultural fields for the years 1987, 2000 and 2017. In Ethiopia, the Ministry of Agriculture and Rural Development (MOARD) is responsible for large-scale land deals with foreign and local investors [8]. Since 2008, MOARD has been transferring investment lands ranging from 500 hectares (ha) to a maximum of 5000 ha [33] which is far larger than small-scale farm land size, which is less than 2 ha [34]. Gambella agricultural office documents and LU/LC maps of the study area were used also to differentiate large-scale commercial farmland from small-scale farmland. In addition to this, in 2017, types of crop-growing by large-scale farmers were identified based on the fieldwork survey (in-situ data) and using the high-resolution (Sentinel 2a) satellite images.

## 3 Results

### 3.1 LULCC classification

As a first result, the accuracy assessment of the supervised LULCC classifications shows an overall accuracy of 82.7% for 1987, 88.4% for 2000 and 94.4% for 2017. The Kappa coefficients for 1987, 2000 and 2017 are 0.78, 0.86 and 0.93 respectively. The higher accuracy of Sentinel is related to the higher resolution of the images. These high values for the selected classes in Table 2 allow for a pixel-wise analysis of the changes in these LU/LC classes over time. These are listed in Table 6. During the last three decades (1987, 2000 and 2017), the gross changes in area coverage varied from one LU/LC class to another. Barren or sparsely vegetated land and farmland class experienced the biggest increase, and tropical grasslands underwent the largest decrease in area coverage, as shown in Table 6.

**Table 6:** Total area coverage/net change and percentage change occurring between the years 1987, 2000 and 2017 for the classified LU/LC categories.

LU/LC class	Area (1987)		Area (2000)		Area (2017)		1987–2000 Net area change (km^2^)	2000–2017 Net area change (km^2^)
	km^2^	%	km^2^	%	km^2^	%		
Water bodies	11	0.04	53	0.21	50	0.20	**+42**	**-3**
Artificial areas	33	0.12	431	1.69	218	0.85	**+398**	**-213**
Farmland	2,121	8.31	2,095	8.21	2,694	10.55	**-26**	**+599**
Forest land	4,188	16.41	4,524	17.73	3,948	15.50	**+336**	**-576**
Tropical grasslands	13,716	53.74	10,704	41.94	9,112	35.70	**-3,012**	**-1,592**
Wetland vegetated area	2,056	8.05	6,435	25.21	5,953	23.32	**+4,379**	**-482**
Barren or sparsely vegetated land	3,396	13.30	1,279	5.01	3,546	13.90	**-2,117**	**+2,267**
Total	**25,521**		**25,521**		**25,521**			

Bold fonts are used to emphasis the magnitude of net area changes between the years 1987–2000 and 2000–2017.

**Fig. 2:**
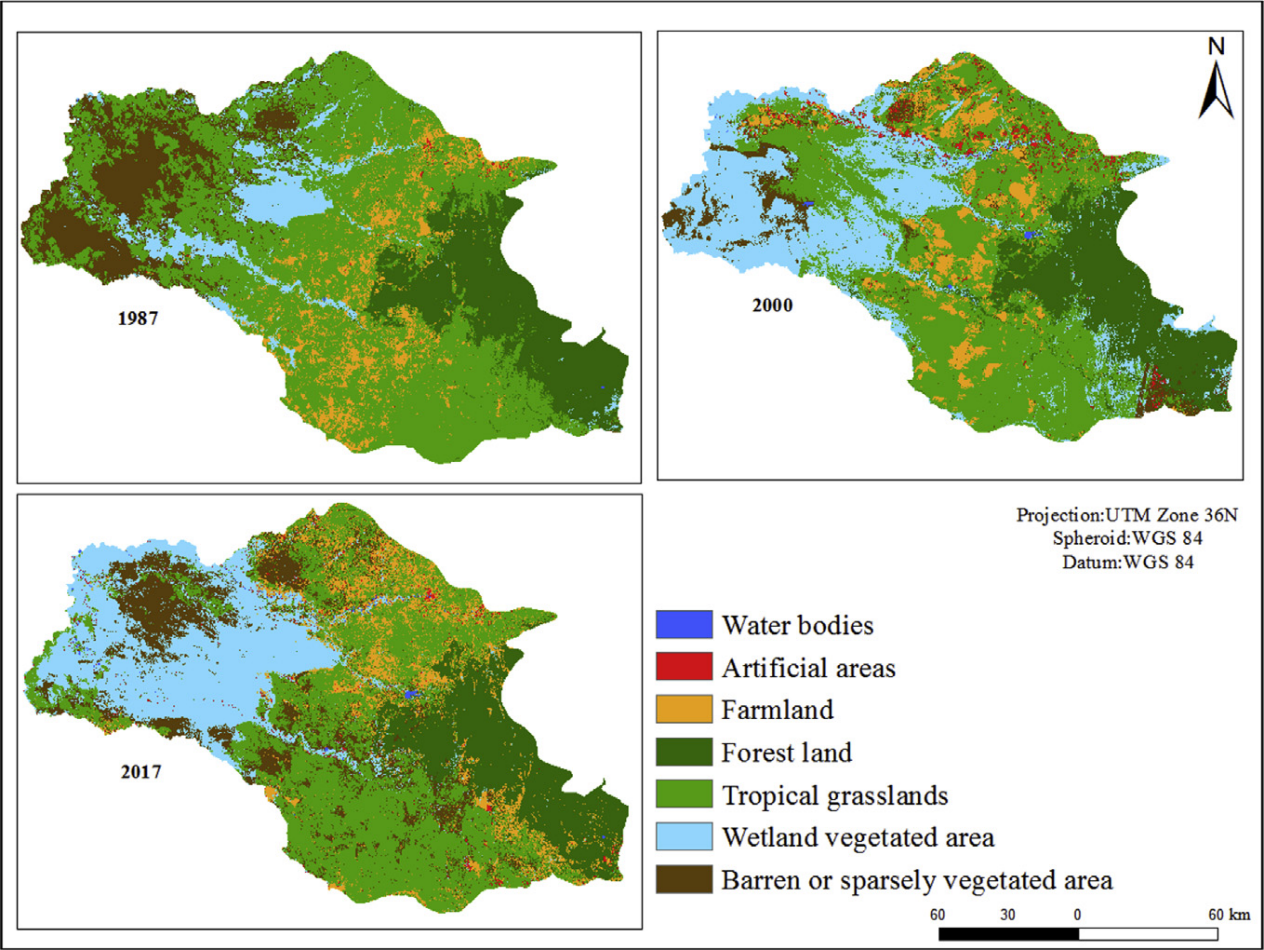
Spatial distributional pattern of LULCC of the Gambella Region, Landsat 5 for 1987 (left), Landsat 5 for 2000 (right) and Sentinel 2A for 2017 (bottom left) supervised classification.

The total area percentage of each class in 1987, 2000 and 2017 shows that tropical grasslands had the largest share in 1987, representing 53.74% (13,716 km^2^) of the total LU/LC categories assigned. This class underwent a major shift and was reduced to 41.94% (10,704 km^2^) and 35.70% (9,112 km^2^) in 2000 and 2017 respectively. The other class which faced a decline during the study period was forest land. The area of this class in 1987 was 16.41% (4,188 km^2^) of the total area and in 2000 it showed a slight increase to 17.73% (4,524 km^2^). However, by 2017 it had reduced to 15.50% (3,948 km^2^). The major LULCC was also observed in the wetland vegetated area. Its share increased from 8.05% (2,056 km^2^) in 1987 to 25.21% (6,435 km^2^) in 2000, although it had decreased slightly to 23.32% (5,953 km^2^) by 2017. The wetland area class expansion, which had been observed in the last 30 years, was not due to rainfall variation. In fact, according to World Meteorological Organisation (WMO) data, the average annual rainfall in the Gambella region during 1987 was higher than in 2000 and 2017 [35]. Therefore, wetland vegetated area cover in 1987 was lower compared to 2000 and 2017 due mainly to the construction of the Alwero Dam, which started in 1984 and led to the diversion of the Alwero river flow until 1992, reducing the water flow downstream [36]. On the other hand, agricultural area showed a slight decrease from 8.31% (2,121 km^2^) in 1987 to 8.21% (2,095 km^2^) in 2000, although by 2017, it had increased to 10.55% (2,694 km^2^). The change in the water class was not very significant, although it did increase during the study period. The share of the total area was 0.04% (11 km^2^) in 1987, 0.21% (53 km^2^) in 2000 and 0.20% (50 km^2^) in 2017. On the other hand, the share of barren or sparsely vegetated land cover shows significant changes, decreasing from 13.30% (3,396 km^2^) in 1987 to 5% (1,279 km^2^) in 2000. Despite this, barren or sparsely vegetated land had increased by 2017 and now covers 13.90% of the total area (3,546 km^2^). The supervised classification results are illustrated below in Fig. 2.

#### 3.1.1 LU/LC area percentage change (1987–2000) and (2000–2017)

The area percentage change shows the extent to which the different LU/LC class areas contribute to net gains (the amount of area that converts into a given class between two dates) and/or net losses (the amount of area that converts out of a given class between two dates) between 1987 and 2000. Artificial areas, wetland vegetated areas and forest land increased by 1206%, 213% and 8% respectively. Similarly, open water bodies increased by 382% in 2000. This increase in the share of the water class was mainly related to the Alwero Dam. The dam reservoir now stores 74,600 million cubic metres of water [37]. Originally, the dam was planned to irrigate a state cotton farm; however, that never materialised [36]. Lately, however, one of the largest farm companies (Saudi Star) constructed around 30 km of channels to transport the water from the dam to irrigate large rice fields. On the other hand, barren land, tropical grassland and farmland decreased by 62%, 22% and 2% respectively.

The area percentage change between 2000 and 2017 showed that most of LU/LC class area percentage changes had decreased. Artificial areas, tropical grasslands, forest land and wetland vegetated area decreased by 50%, 15%, 13% and 8% respectively. In contrast, barren land and farmland increased by 177% and 29% compared to 2000. The expansion of large-scale farming investment in the Gambella region is one of the key factors for the farmland area percentage change. In the past two decades, water cover remained constant and did not show any significant change.

### 3.2 LULCC detection for the years 1987, 2000 and 2017

A post-classification comparison of the changes in the classified distribution of LU/LC categories was carried out in order to produce change maps with the purpose of gaining insight into the spatial patterns of change between the selected years. It also documents, spatially, the amount of conversion from a particular LU/LC to another LU/LC category [28]. The ‘from-to’ information of the Gambella region LU/LC maps is shown in Tables 7 and 8.

**Table 7:** LULCC confusion matrix in km^2^ of 1987 to 2000.

LU/LC type	Water bodies		Artificial areas		Farmland		Forest land		Tropical grasslands		Wetland vegetated area		Barren or sparsely vegetated area		Total 2000	Gain in 2000 (km^2^)
	Area Km^2^	%	Area Km^2^	%	Area Km^2^	%	Area Km^2^	%	Area Km^2^	%	Area Km^2^	%	Area Km^2^	%	Area Km^2^	
Water bodies	7	64	0	0	2	0	1	0	27	0	7	0	10	0	53	46
Artificial areas	0	0	11	33	56	3	10	0	245	2	18	1	91	3	431	420
Farmland	0	0	2	6	342	16	2	0	1,617	12	18	1	114	3	2,095	1,753
Forest land	1	9	0	0	33	2	3,876	93	464	3	140	7	10	0	4,524	648
Tropical grasslands	0	0	17	52	1,567	74	72	2	7,963	58	256	12	830	24	10,704	2,741
Wetland vegetated area	3	27	3	9	98	5	183	4	2,782	20	1,553	76	1,813	53	6,435	4,882
Barren or sparsely vegetated area	0	0	0	0	23	1	44	1	618	5	64	3	528	16	1,279	751
Total 1987	11		33		2,121		4,188		13,716		2,056		3,396		25,521	
Loss in 1987 (km^2^)	4		22		1,779		312		5,753		503		2,868

**Table 8:** LULCC confusion matrix in km^2^ of 2000 to 2017.

LU/LC Type	Water bodies		Artificial areas		Farmland		Forest land		Tropical grasslands		Wetland vegetated area		Barren or sparsely vegetated area		Total 2017	Gain in 2017 (km^2^)
	Area Km^2^	%	Area Km^2^	%	Area Km^2^	%	Area Km^2^	%	Area Km^2^	%	Area Km^2^	%	Area Km^2^	%	Area Km^2^	
Water bodies	31	58	0	0	0	0	1	0	0	0	14	0	4	0	50	19
Artificial areas	2	4	25	6	7	0	22	0	67	1	78	1	17	1	218	193
Farmland	5	9	130	30	292	14	379	8	1,287	12	444	7	157	12	2,694	2,402
Forest land	2	4	7	2	0	0	3,691	82	37	0	174	3	37	3	3,948	257
Tropical grasslands	2	4	131	30	1,381	66	110	2	6,207	58	950	15	331	26	9,112	2,905
Wetland vegetated area	10	19	59	14	39	2	225	5	1,024	10	4,175	65	421	33	5,953	1,778
Barren or sparsely vegetated area	1	2	79	18	376	18	96	2	2,082	19	600	9	312	24	3,546	3,234
Total (2000)	53		431		2,095		4,524		10,704		6,435		1,279		25,521	
Loss in 2000 (km^2^)	22		406		1,803		833		4,497		2,260		967			

#### 3.2.1 Change detection between 1987 and 2000

We found significant conversions from one land cover category to another. Tropical grasslands were mainly converted to wetlands (2,782 km^2^) and farmland (1,617 km^2^). There were also significant conversions of tropical grasslands to artificial areas (245 km^2^). On the other hand, 830 km^2^ of barren land was converted into tropical grasslands. About 183 km^2^ and 44 km^2^ of what was forest land in 1987 was converted to wetlands and barren lands respectively. Around 1,813 km^2^ of barren land was also converted into wetland vegetation. Because of the largely unsuccessful resettlement programme during the 1990s, farmland that had been allocated by the Derg government in 1984–1985 was abandoned by farmers, who walked thousands of miles in order to return to their native regions [38]. As a consequence of the rebound of the resettlement programme and the following depopulation of large areas, 1,567 km^2^ of the newly established farmland became tropical grasslands, and 98 km^2^ became wetland vegetated areas.

#### 3.2.2 Change detection between 2000 and 2017

Change detection between 2000 and 2017 shows that the major observed change was from tropical grasslands to barren land (2,082 km^2^) and wetland vegetated areas (1,024 km^2^). Large areas of forests were converted to farmland, wetland vegetated areas, tropical grasslands and barren land (379 km^2^, 225 km^2^, 110 km^2^ and 96 km^2^ respectively). Despite the conversion of forests to other LU/LC classes, there was also the conversion of 174 km^2^ of wetland vegetation, 37 km^2^ of tropical grasslands and 37 km^2^ of barren land to forests. In recent times, the Ethiopian government has allocated part of the wetland vegetated area to large-scale commercial investors with the clear intention of draining water from the wetlands for irrigation purposes. As a result, around 444 km^2^ of wetland vegetated area was changed into farmland area. In addition, there was a significant conversion to farmland from tropical grasslands (1,287 km^2^). Table 8 shows that what was barren land (157 km^2^) and an artificial area (130 km^2^) in 2000 has now been converted into farmland in 2017. In contrast, due to the VVS programme in the Gambella region in the 2000s, around 1,381 km^2^ of farmland was abandoned and converted to tropical grasslands.

### 3.3 Farmland expansion and farming intensity

**Fig. 3:**
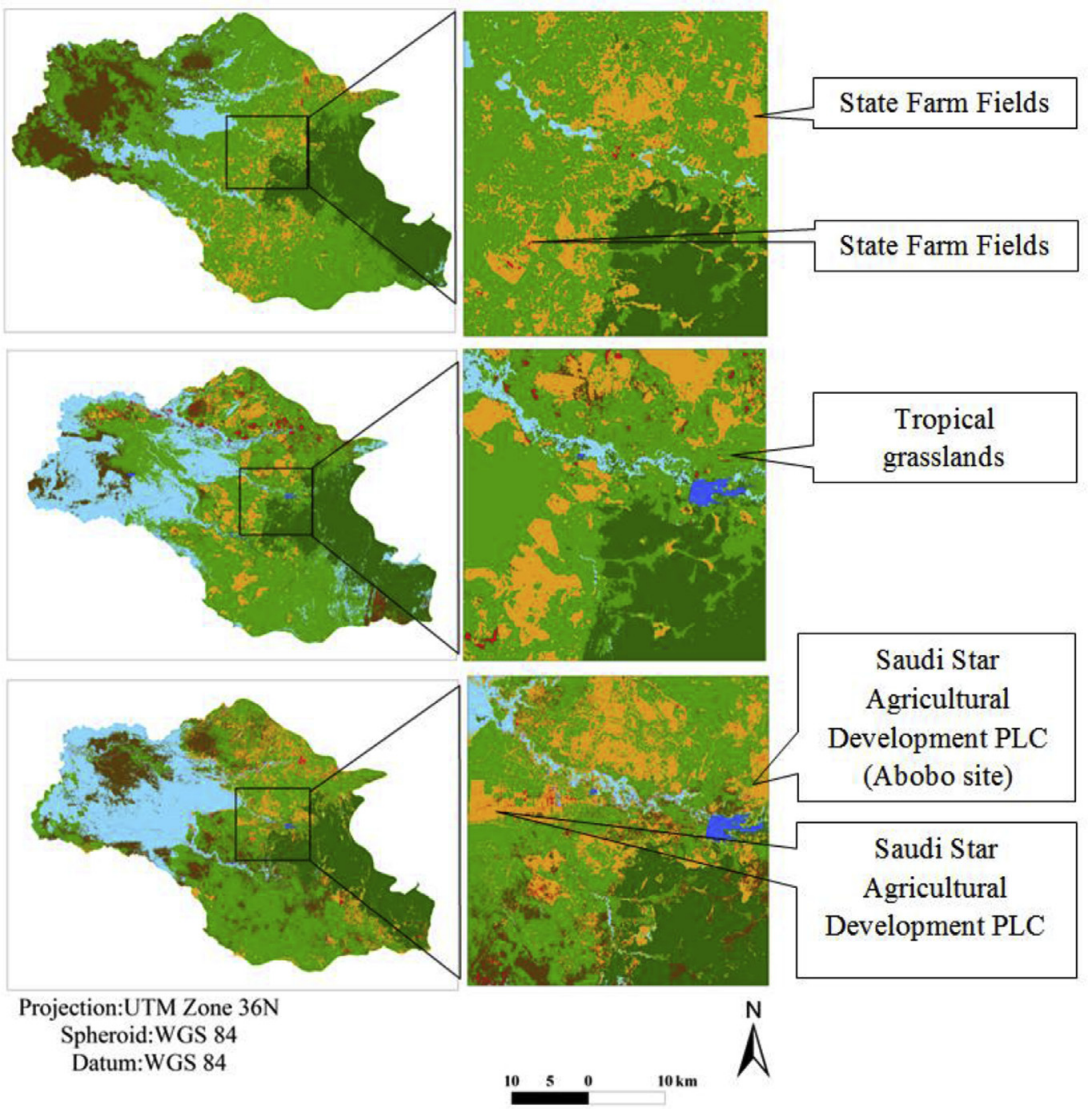
Supervised image classification identified farmland expansion in the central part of Gambella region during 1987, 2000 and 2017. State-farm fields in Landsat 1987 (top), state-farm fields which are banded and turn into grasslands shown in Landsat 2000 (middle) and State-farm fields turned into large-scale farm fields from Sentinel 2a satellite image 2017 (bottom).

This chapter takes a closer look at the expansion of farmland and its related division into different farming intensities. It is aimed at quantifying the change in smallholder and large-scale commercial farms separately. Whereas area expansion can easily be quantified with the change analysis documented in Chapter 3.2, the classification results say nothing about farming intensity. Agricultural area increased by a total of 573 km^2^, or 27% of the area change in the Gambella region between 1987 and 2017. In total about 202 km^2^ of cultivated area was identified as large-scale commercial farmland in 1987 and these large-scale farm fields had been under the control of state farms, as shown in Fig. 3. However, by 2000, this large-scale state farming had been completely abandoned, and as a result, its land use had decreased to zero.

In 2017, based on the fieldwork survey (in-situ data) and using Sentinel 2a satellite images, a total area of 746 km^2^ of large-scale farm fields were identified and then this large-scale farm fields were masked from Sentinel 2a satellite images and classified, of which 415 km^2^ were rice, 35 km^2^ were cotton, 88 km^2^ sesame, and the remaining 208 km^2^ were covered by various types of cash crops. In total, large-scale commercial farmland has increased by 269% in the last three decades in the study area.

On the other hand, in 1987, around 1,919 km^2^ of farmland was cultivated by small-scale farmers. This area increased to about 2,095 km^2^ by 2000. This shows that small-scale farmland in the region had increased between 1987 and 2000 by a total of 176 km^2^ or 9.2%. In 2017, the total cover of small-scale farmland was 1948 km^2^, which is a decrease of 147 km^2^ or 7%. After 2011, the land which was aquired by smallholders had been given over to large-scale farm land or investors. As a result, the total land holding size of small-scale operations decreased in 2017. Small-scale farmers are subsistence farmers and mainly cultivate cereal crops such as sorgum, millet and maize.

### 3.4 Gambella National Park LULCC

**Fig. 4:**
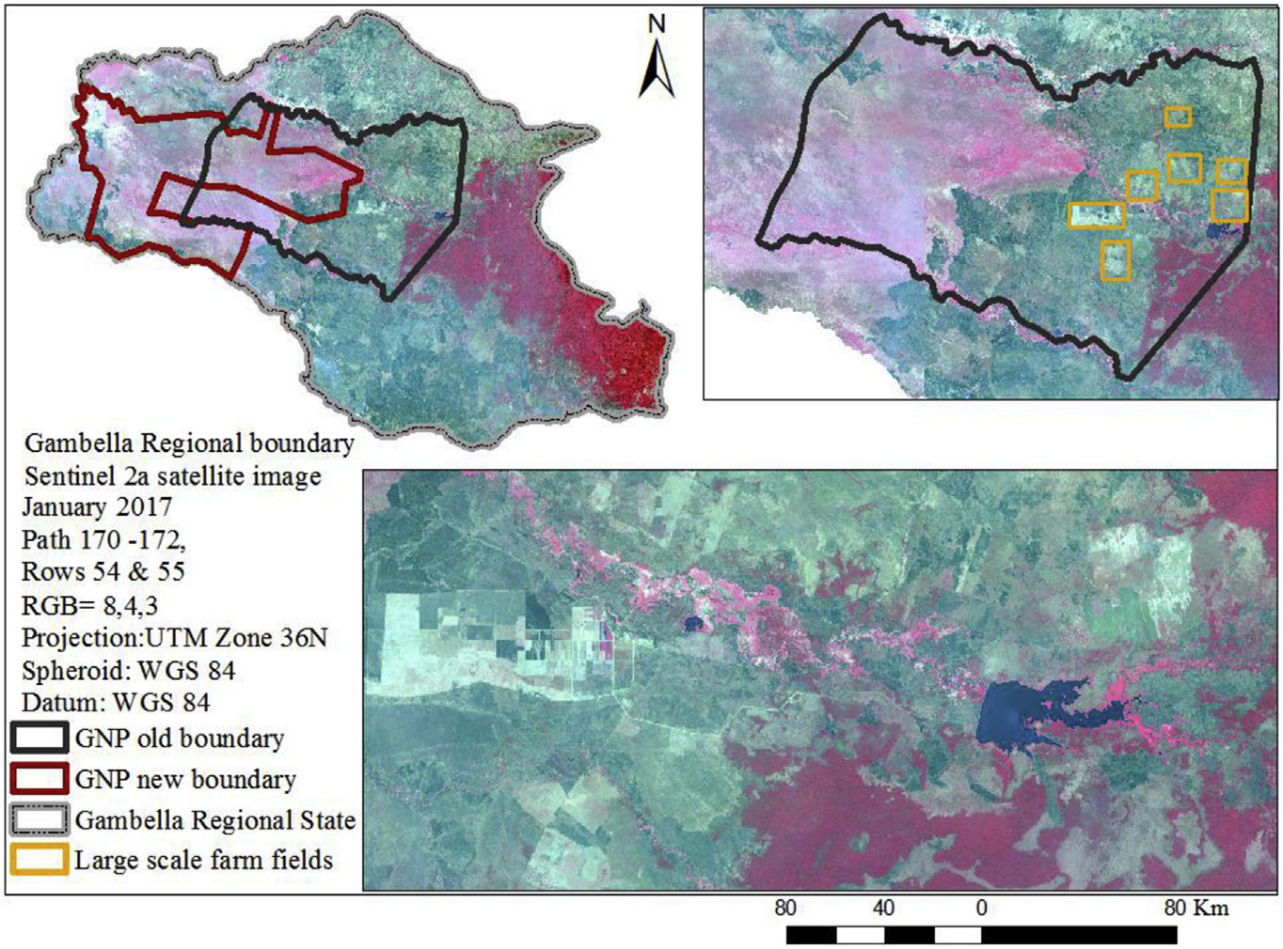
The old and new proposed Gambella National Park boundary (top left) and rectangles enclosing large-scale commercial farm fields within the present GNP boundary from Sentinel 2a satellite image 2017 (top right) and zoomed out large-scale commercial farm fields within the old GNP boundary from Sentinel 2a satellite image 2017 (below).

Around 5,700 km^2^ of the Gambella region is covered by Gambella National Park (GNP). However, currently there is new boundary which covers around 4,350 km^2^ and is shifted towards the south western parts of the region (as shown in Fig. 4). It also means that the GNP would lose about 1,500 km^2^ or about 25% tropical grassland cover of its area.

**Fig. 5:**
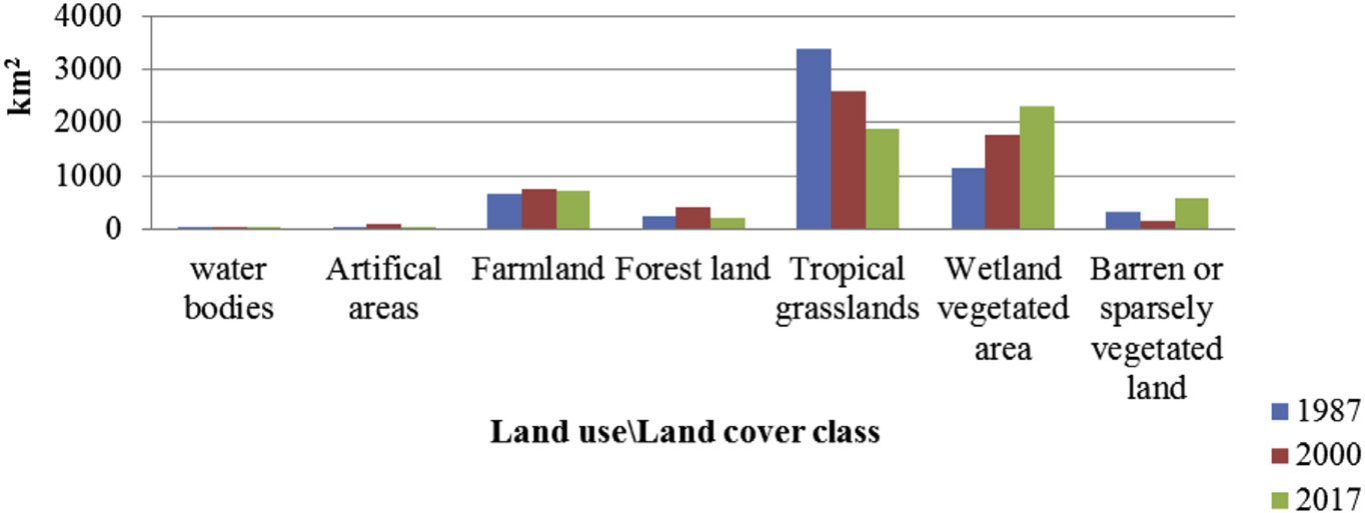
Changes in the composition of Gambella National Park LU/LC categories in the old GNP boundary (1987, 2000 and 2017).

**Fig. 6:**
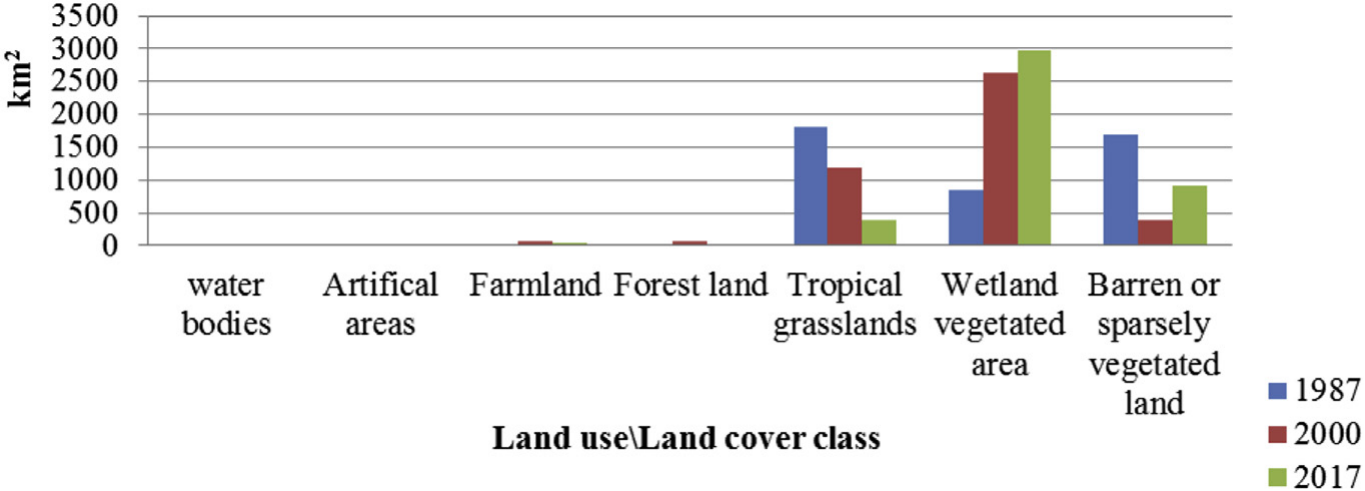
Changes in the composition of Gambella National Park LU/LC categories in the new proposed boundary (1987, 2000 and 2017).

Changes in LU/LC categories within the old GNP showed that areas of approximately 672 km^2^, 761 km^2^ and 725 km^2^ were covered by farm fields in 1987, 2000 and 2017 respectively. As shown in Fig. 5, forest cover has increased from 250 km^2^ in 1987 to 394 km^2^ in 2000. However, by 2017, forest cover had decreased to 211 km^2^. In addition, tropical grasslands, which covered an area of 3,385 km^2^ in 1987, decreased to 2,584 km^2^ in 2000 and 1,887 km^2^ in 2017. Man-made structures, such as settlements, expanded from about 7 km^2^ in 1987 to 101 km^2^ in 2000, although they have since decreased to 37 km^2^ by 2017. Within the new proposed GNP boundary, however, both agricultural activities and artificial areas are to be restricted. Abandoned farming activities and artificial areas within the new GNP will have the greatest chance of being incorporated into the GNP ecosystem. In the new GNP boundary (as illustrated in Fig. 6) the farmland cover was 19 km^2^ and 60 km^2^ in 1987 and 2000 respectively. However, in 2017 the farmland cover had decreased to 51 km^2^. In addition, in 2017 within this new proposed boundary, 920 km^2^ are under barren or sparsely vegetated land, while 378 km^2^ are under tropical grasslands and 2,970 km^2^ are wetland vegetated areas. In general, the expansion of wetland vegetated areas in the new proposed boundary has had a positive impact on wildlife conservation and on the ecological management of the park [39].

## 4 Discussion

The supervised classification of multi-temporal satellite images is an effective tool to quantify current LU/LC, as well as to detect changes in a changing environment [32]. The study reveals that in the last three decades, tremendous LULCC have been observed in the Gambella region. These LULCC have been caused by a mixture of climatic factors and strong human interference and have impacts on both the natural environment and on people's livelihoods.

The observed trend of expanding agricultural area in Gambella region between 1987 and 2017 is supported by Ethiopian government, since large-scale land investment is an important part of its development strategy [40]. In the 1980s, the previous Ethiopian government, Derg, introduced large state farms in the region. However, in 1991, the state farms collapsed and the farmland was abandoned after the abolition of the Derg government [41]. Since the mid-2000s, however, the Ethiopian People's Revolutionary Democratic Front (EPRDF) have handed over previously state-owned farmlands to foreign companies [42]. As a result, large-scale farming activities have expanded rapidly in the Gambella region within a short period of time.

According to the Ethiopian Investment Agency's official report, from 2004 to 2015 around 185 investors were granted licence to invest in the Gambella region, out of which 22 were foreign investors and the remainder were local investors [43]. By 2008, in Gambella alone, MOARD made around 47% or 1.1 million ha of suitable farmland available to large-scale farm companies. Nevertheless, MOARD asserted that while some of the investors are operating their lands wisely the majority are leaving their lands barren and uncultivated. Large-scale commercial farmland expansion threatens the land rights and livelihoods of indigenous communities, and nowadays their land is given for cash crop/monocrop production. For instance, in 2012, the Ethiopian government removed/relocated more than 70,000 indigenous people as a result of large-scale commercial farming activities [12, 44]. This relocation/resettlement has had a negative impact on small-scale farmland expansion. In the past three decades, small-scale farmland increased by just 29 km^2^, or 1.5%. Although the Central Statistics Agency (CSA) report indicated that population between 2007 and 2015 in Gambella region was increasing at around 4.15% per year, small-scale farmland expansions to meet short-term survival rates are insignificant. According to a World Bank report [34], the average land holding size per family in the study area is 1.4 ha, which is less than the average standard land holding size of 2 ha, recommended by the Food and Agriculture organization (FAO) [45]. Land holding size, land/property rights and food insecurity are therefore the major concerns of small-scale farmers [45, 46]. Since the 2010s, land conflicts and clashes between the two major ethnic groups (Anyuaa and Nuers) have largely increased in the region over farmland [47].

The Oakland Institute (2011) conducted a study on the impact of large-scale agricultural investment on local people's livelihoods and LULCC in Gambella Regional State. The findings reveal that investors cleared huge areas of forest by practising the slash-and-burn farming system in their fields [48]. This is the greatest threat to forest cover, which is the main source of food for the hunter-gatherer indigenous peoples [13]. In 1987, the total forest cover was 4,188 km^2^; however, in 2000 there was neither state nor large-scale farming investment in the region. As a result, the forest cover increased to 4,524 km^2^. This study also confirmed that the forest cover and large-scale agricultural investment has an indirect/inverse relationship. For instance, forest cover declined when large-scale farm investment was restarted in the region in the mid-2000s and this implies that about 576 km^2^ of forest land has disappeared between 2000 and 2017. Recent statistics also indicate that the annual deforestation rate in Ethiopia in general is about 1,410 km^2^ per year [49] due to farmland expansion. With this pace of deforestation, the unique forest cover of the Gambella region, with its rich biodiversity, will have disappeared by approximately 2133. This would have a significant impact on the LU/LC ecology and biodiversity through the whole Gambella region.

On the other hand, in 2003, the FDRE established a revitalisation of population relocation strategies of the resettlement and villagisation (R&V) programme. This programme resurrected the problems last seen during the Derg regime. As a strategy of transforming the livelihood of settlers and ensuring food security by providing socioeconomic and infrastructural delivery, it is a voluntary principle through an intra-regional approach. Since the mid-2000s, scattered households have been collected together into selected nucleated villages in order to improve their access to social, economic and administrative services in the Gambella region. As a result of this, farmers had to leave their ancestral farmlands and relocate to villages. Thus, according to our findings, about 1,381 km^2^ of farmland has been abandoned and re-converted into tropical grasslands because of the R&V programme. In the past, a poorly planned resettlement programme leading to uncontrolled encroachments has caused great damage to the vegetation composition and structure of the area [11]. For instance, resettlement sites were established in the 1980s by clearing dense natural forests.

Our results show that there are strong human interferences within the old boundaries of the national park, as seen e.g. by the farming activities and the decrease of forest land, while within the new boundaries, almost no farming activities were observed in 2017 and also less artificial areas exist in the new boundaries. However, the new boundaries of GNP contain less forest land than in the old boundaries. Besides LU change, we identified large areas of LC change within both, the old and the new boundaries of the GNP. Over 1.498 km^2^ of natural grassland has been transformed mainly into wetlands over the last decades. Several studies support our findings within the GNP and identify possible reasons for this development. According to [39] the changes are not only driven by natural processes, but mainly due to human interferences, such as unsustainable utilization of natural resources such as deforestation, commercial farm expansion, and settlement/encroachment, and wild fires. Since the 1990s, the government's land allocations to external agribusiness investors in and around the GNP has threatened the livelihoods of local communities [50], and currently the pristine areas of the park are shrinking and suffering widespread damage to plant species and wild animals [51]. The clearing of land and large-scale deforestation has increased competition for land between investors and local farmers. All these syndromes contradict the idea of a protected area and caused social and economic hardship [11].

Since 2015, the Ethiopian government has proposed a new park boundary to make the park more suitable for wildlife conservation and ecosystem management [52]. As result, the government is restricting encroachment/settlement and large-scale/commercial farming activities within the new GNP boundary. Recently, the national park has also had the support of foreign-funded programmes, which is taking part in the setting up of a land use plan for the Gambella region [53].

## 5 Conclusion

From the overall research findings, some important conclusions can be drawn. Remote sensing has an outstanding value as an independent and objective source of information on LULCC. In the Gambella resettlement program, demographic growth and large-scale land investment has caused social and environmental impacts, as forests are cleared and communities are displaced, or lose access to farmland, forest or water resources. Particularly, large-scale land investment without proper environmental impact assessments and unplanned resettlement programmes were the cause of enormous LULCC in the Gambella region. Failed resettlement programmes in the 1980s and R&V in the mid-2000s led to the re-establishment of grasslands that are important for a rewilding process in the region, which is unique in Ethiopia. This rewilding process in the Gambella region is able to allow indigenous plant species to grow on their own and produce new generations without human intervention.

The results of this research revealed the existence of significant LULCC over the last 30 years. The observed changes varied from one LULCC category to another. This study showed that the expansion of large-scale commercial farmland increased in 2017 compared to 1987. The expansion of small-scale farmland between 1987 and 2017, on the other hand did not show any significant change, and it is less than 2% area change unless one would expect a higher increase to a massive population growth. This shows the dominant influence of commercialisation on agriculture in the region. In addition, barren land has increased considerably during the last two decades. On the other hand, tropical grassland and forest areas are declining at a rapid pace.

The GNP, which is the nation's largest national park ecosystem has also been affected by LULCC. The conversion of tropical grasslands and forest land to large-scale farmland has caused varied and extensive environmental degradation in the GNP, and major negative outcomes for local people's livelihood. For instance, the establishment of the new park boundaries resulted in a loss of large parts of the wetland vegetated area and water bodies. Identifying the complex interaction between changes and its drivers over time is significant in determining future change, setting up decision-making mechanisms and constructing alternative scenarios. Therefore, in future, sustainable development LU/LC has to be monitored at regular intervals and an integrated LU policy is required.

In general, this study advocates the use of multi-temporal and high-resolution satellite data to detect and assess changes in LU/LC comprehensively. Only local field work and the compilation of additional sources can enable the observed changes to be put into a larger perspective, and to be able, finally, to understand the LU/LC dynamics in the Gambella region. This is a necessary prerequisite to formulating successful LU strategies required for the appropriate and sustainable development of the study area.

To sum up, this paper has documented LULCC in the Gambella region of Ethiopia. It quantifies the changes and illuminates reasons and effects. The Gambella region is among the least developed and the most fertile regions of Ethiopia. It has large natural reserves in terms of pristine forests, wetlands, tropical grasslands and biodiversity. At the same time, its future development potentials are large and based on solid indicators like available rainfall, fertile soils and accessible land. Although all documented LULCC during the last decades point towards the usual conversion of natural land into human utilisation, one may also conclude that Gambella region is still far away from a terminal stage of human interference. This opens up the chances to develop and implement policies to ensure the sustainable future development of the Gambella region. It should enforce non-interference into the new GNP boundary in order to protect the remaining pristine areas, develop smallholder agriculture towards larger yields to raise income and at the same time preserve natural areas of wetland and grassland. In addition, large-scale farming should be slowed down and should demonstrate sustainable operations with respect to, e.g. erosion and pollution.

## Declarations

### Author contribution statement

Azeb W Degife: Conceived and designed the experiments; Performed the experiments; Analyzed and interpreted the data; Contributed reagents, materials, analysis tools or data; Wrote the paper.

Florian Zabel, Wolfram Mauser: Conceived and designed the experiments; Analyzed and interpreted the data; Wrote the paper.

### Funding statement

This research did not receive any specific grant from funding agencies in the public, commercial, or not-for-profit sectors.

### Competing interest statement

The authors declare no conflict of interest.

### Additional information

No additional information is available for this paper.
